# Inferring Regulatory Networks From Mixed Observational Data Using Directed Acyclic Graphs

**DOI:** 10.3389/fgene.2020.00008

**Published:** 2020-02-07

**Authors:** Wujuan Zhong, Li Dong, Taylor B. Poston, Toni Darville, Cassandra N. Spracklen, Di Wu, Karen L. Mohlke, Yun Li, Quefeng Li, Xiaojing Zheng

**Affiliations:** ^1^Department of Biostatistics, University of North Carolina at Chapel Hill, Chapel Hill, NC, United States; ^2^Department of Pediatrics, University of North Carolina at Chapel Hill, Chapel Hill, NC, United States; ^3^Department of Genetics, University of North Carolina at Chapel Hill, Chapel Hill, NC, United States; ^4^Department of Oral and Craniofacial Health Science, University of North Carolina at Chapel Hill, Chapel Hill, NC, United States

**Keywords:** regulatory network, directed acyclic graphs, mixed observational data, continuous and categorical variables, causal regulatory pathways

## Abstract

Construction of regulatory networks using cross-sectional expression profiling of genes is desired, but challenging. The Directed Acyclic Graph (DAG) provides a general framework to infer causal effects from observational data. However, most existing DAG methods assume that all nodes follow the same type of distribution, which prohibit a joint modeling of continuous gene expression and categorical variables. We present a new mixed DAG (mDAG) algorithm to infer the regulatory pathway from mixed observational data containing both continuous variables (e.g. expression of genes) and categorical variables (e.g. categorical phenotypes or single nucleotide polymorphisms). Our method can identify upstream causal factors and downstream effectors closely linked to a variable and generate hypotheses for causal direction of regulatory pathways. We propose a new permutation method to test the conditional independence of variables of mixed types, which is the key for mDAG. We also utilize an *L*_1_ regularization in mDAG to ensure it can recover a large sparse DAG with limited sample size. We demonstrate through extensive simulations that mDAG outperforms two well-known methods in recovering the true underlying DAG. We apply mDAG to a cross-sectional immunological study of *Chlamydia trachomatis* infection and successfully infer the regularity network of cytokines. We also apply mDAG to a large cohort study, generating sensible mechanistic hypotheses underlying plasma adiponectin level. The R package mDAG is publicly available from CRAN at https://CRAN.R-project.org/package=mDAG.

## Introduction

Identification of differentially expressed genes associated with disease has become an instrumental approach, but with only limited success in mechanistic discovery, partly due to the fact that current methods based on fold-change focus only on a single gene. Co-expression network analysis (Oldham et al., 2006; Chen, 2012; Hawrylycz et al., 2012), an approach that constructs networks of genes that tend to co-activate among a group of samples, provides a connectome of gene interaction. (Zhuang et al., 2016) proposes a more general class of undirected graphical models that can handle mixed types of variables. However, the undirected graphical model by itself cannot reveal disease causality. There is a critical need to understand regulatory pathways for discovery of therapeutic targets and disease mechanisms.

A few approaches have been proposed in recent years to estimate regulatory networks/pathways. iPoint was proposed by Atias and Sharan (2013) to infer a compact subnetwork that connects the source of the response (*anchor* genes) to the targets of the response (*terminal* genes) while optimizing local (individual path lengths) or global (likelihood) aspects of the subnetwork to solve the “anchor” reconstruction problem. The input of iPoint requires a single *anchor* gene and a list of *terminal genes*. PINE was proposed by Wilentzik and Gat-Viks (2015) to identify the particular pathways by which DNA variants perturb the signaling network. It requires prior established biological knowledge of how the stimulations affect gene expression and existence of multiple stimulation conditions. TieDie was proposed by Paull et al. (2013) to infer regulatory pathways linking genomic events (e.g. mutated genes) to transcriptional changes by a heat diffusion strategy. However, TieDie assumes that mutations necessarily lead to loss of function. All these methods assume prior knowledge of particular biological networks/pathways or functions.

Over the past few years, there has been a growing interest in utilizing directed acyclic graphs (DAG), which do not require any prior biological knowledge, to infer directional relations in a regulatory network in a large variety of disciplines such as biology, neuroscience, and psychology (Friedman et al., 2000; Huang et al., 2010; Borsboom and Cramer, 2013). The logical basis of such graphical models is the conditional independence structure of the underlying probability distributions of data. We propose to jointly model the probability distribution of mixed data composed of continuous variables (e.g., expression of proteins or genes) and discrete variables (e.g., categorical disease outcomes or single nucleotide polymorphisms) by DAG.

There are three types of methods to estimate a DAG (Nagarajan et al., 2013): constraint-based methods, score-based methods, and hybrid methods. The constraint-based methods learn a DAG by exploiting the conditional independence constraints in the observational distribution. The most prominent example of such methods is the PC algorithm (Spirtes et al., 2000). This algorithm first estimates the skeleton of the underlying DAG, and then adds orientations to the skeleton based on a set of edge orientation rules (Meek, 1995). The CPC-stable algorithm (Colombo and Maathuis, 2014) improves the PC algorithm by resolving the order-dependence issue in the determination of the skeleton. A more recent constraint-based method (Tsagris et al., 2018) proposes a symmetric conditional independence tests based on likelihood-ratio test and combines it with the existing constraint-based methods (e.g. PC algorithm) to estimate a DAG. The score-based methods (Chickering, 2002) learn a DAG by a greedy search for the optimal score of the goodness-of-fit of the estimated DAG. The hybrid methods (Nagarajan et al., 2013) learn a DAG by integrating the constraint-based and the score-based methods. An example is the Max-Min Hill-Climbing (MMHC) algorithm (Tsamardinos et al., 2006), which applies the Max-Min Parents and Children algorithm to obtain the skeleton and the Hill Climbing greedy search algorithm to orient edges in the skeleton. Another example is the causalMGM algorithm (Sedgewick et al., 2016; Sedgewick et al., 2017), which firstly estimates an undirected graph and then uses PC-stable or CPC-stable for orientation. The first step modifies the mixed graphical model method (Lee and Hastie, 2015) by using different penalty functions for different edge types. The second step uses a likelihood-ratio test to test the conditional independence in order to use the PC-stable or CPC-stable algorithm for edge orientation. Based on our experience, such an orientation method is not as efficient as score-based method, which is used in our algorithm.

However, most of these methods assume that all variables are of the same type. For example, the Gaussian graphic model assumes that the joint distribution of all variables is multivariate normal. Therefore, these methods cannot be directly applied to infer the causal relationship between continuous measurements, such as protein or gene expression, and the categorical variables, such as categorical traits or single nucleotide polymorphisms (SNPs). To this end, we propose a mixed DAG method (mDAG) that accommodates data of different types. We assume the joint distribution of all variables follow a pairwise Markov random field, which ensure that the conditional distribution of one graph node on all other nodes either follow a Gaussian distribution or a multinomial distribution. Thus, it enables joint modeling of continuous and categorical variables. We demonstrate the efficacy of our method through extensive simulations and apply it to a study of human cytokines associated with chlamydial susceptibility to infer cytokines with causal effects on a categorical disease phenotype. We also show that our method can identify gene expression levels that mediate the effect of genetic variants on traits.

## Materials and Methods

### Definitions and Preliminaries

We first introduce a few key concepts in the DAG theory. A DAG of a vector of random variables *X* = (*X_1_*_,…,_*X_d_*)*^T^* is a directed graph with no cycle, which is denoted by *G* = (*V, E*), where *V* is the set of *d* vertices representing *X*, and *E* is the set of all directed edges. Given a path $X_{i_{0}}\to X_{i_{1}}\to \ldots \to X_{i_{n}}$ in a DAG, $X_{i_{l-1}\;}$ is called a parent of $X_{i_{l}\;}$ and $X_{i_{l}\;}$ is called a child of $X_{i_{l-1}\;}$. The *d* separation set *S* that blocks nodes *i* and *j* is a vertex set that blocks all paths that connect *i* and *j* for either the path that contains at least one arrow-emitting vertex belonging to *S*, or the path that contains at least one collision vertex (a vertex without emitting edges) that is outside *S* and no children of the collision vertex belongs to *S*. In a DAG, the Markov blanket of a node includes its parents, children, and the other parents of all of its children. In an undirected graph, the Markov blanket of a node contains all nodes connecting to itself. The skeleton of a DAG is the undirected graph that results from ignoring the directionality of every edge in a DAG. In order to model the mixed data, we assume the joint distribution of all variables is faithful to a DAG, meaning that for any *i*, *j*∈*V* and any set *S*⊂*V*, *X_i_* and *X_j_* are conditional independent given *X_s_* if and only if node *i* and *j* are *d*-separated by set *S* (Pearl, 2009) and *S* is called the *d*-separation set of node *i* and *j*. In other words, the conditional independence can be read from the DAG. Under the faithfulness assumption, the joint distribution has the Markov property that a node is independent of all other nodes conditional on the Markov blanket. Such an assumption is widely used in Bayesian Network literature, the PC-algorithm (Spirtes et al., 2000), PC-stable and CPC-stable algorithm (Colombo and Maathuis, 2014), and MMHC algorithm (Tsamardinos et al., 2006). Meek (2013) proved that this assumption holds for a variety of Bayesian Network.

To recover the underlying DAG from the mixed data, our method consists of three main steps. First, we use a penalized nodewise maximum likelihood method (Lee and Hastie, 2015) to identify the Markov blanket of each node. Second, we use a modified PC-stable algorithm (Ha et al., 2016) to obtain the DAG's skeleton and its *d*-separation set. Finally, we add orientations to the skeleton using a greedy search algorithm (Tsamardinos et al., 2006). Different from the existing literature, since our data is of mixed types, we propose a new permutation test on the second step to test the conditional independence, which is the key to estimate the skeleton of the DAG for mixed data.

### Identification of the Markov Blanket

We assume the distribution of *X* = (*X_1_,…,X_p+q_*)*^T^* follows a pairwise Markov random field with a density

$$p\left(x;\Theta \right)\propto \exp\left({\text{Σ}}_{s=1}^{p}{\text{Σ}}_{t=1}^{p}-\frac{1}{2}\beta_{st}x_{s}x_{t}+{\text{Σ}}_{s=1}^{p}\alpha_{s}x_{s}+{\text{Σ}}_{s=1}^{p}{\text{Σ}}_{j=1}^{q}\rho_{sj}\left(x_{p+j}\right)x_{s}+{\text{Σ}}_{j=1}^{q}{\text{Σ}}_{r=1}^{q}\phi_{rj}\left(x_{p+j},x_{p+r}\right)\right)$$

where we assume without loss of generality that *X_j_*(*j* = *1*,…*p*) are continuous variables, *X_p+j_*(*j* = *1*,…*q*) are discrete variables, and Θ = (α*_s_*, β*_st_*, ρ*_sj_*, ϕ*_rj_*) for *s,t* = 1,… and *j,r*=1,…*q* are parameters. We assume that the discrete variable X_p+j_ takes a total of *L_j_* values. As shown in (Lee and Hastie, 2015), the conditional distribution of a pairwise Markov random field is either Gaussian or multinomial. Thus, it enables a joint modeling of mixed data. In particular, for a continuous variable *X_j_* its density conditional on all other variables *X_-j_* is given by

$$p\left(x_{j}|x_{-j}\right)=\exp\left\{\frac{1}{\sigma_{j}^{2}}\left[x_{j}x_{-j}^{T}\theta_{j}-\frac{1}{2}{\left(x_{-j}^{T}\theta_{j}\right)}^{2}-\frac{1}{2}x_{j}^{2}\right]-\frac{1}{2}\log2\pi \sigma_{j}^{2}\right\}$$

where *x_-j_* = (*x*_1_,…, *x_j-_*_1_, *x_j+_*_1_,…, *x_p+q_*)*^T^* and *θ_j_*∈*R*^(^*^p^*^+^*^q^*^-1)^ and $\sigma_{j}^{2}\;$ are parameters from the Gaussian distribution. For a discrete variable *X_j_*, its conditional density is given by

$$p\left(x_{j}=i|x_{-j}\right)=\frac{\exp\left\{w_{j}^{0^{\left(i\right)}}+x_{-j}^{T}w_{j}^{\left(i\right)}\right\}}{{\text{Σ}}_{i^{\prime }=1}^{L_{j}}\exp\left\{w_{j}^{0(i^{\prime })}+x_{-j}^{T}w_{j}^{\left(i^{\prime }\right)}\right\}},i\in \left\{1,.,L_{j}\right\}$$

where ${\left(w_{j}^{\left(0\right)},\;\ldots \;w_{j}^{\left(L_{j}\right)}\right)}^{T}\;$ are parameters from the multinomial distribution. In order to recover the Markov blanket, we implement a nodewise penalized generalized linear model (GLM) to perform neighborhood selection for each node (Lee and Hastie, 2015). More specifically, for node *j* we solve a penalized maximum likelihood problem that

$${\hat{\beta }}_{j}=\;\underset{\beta_{j}}{\text{arg min}}\;–{\text{Σ}}_{k=1}^{n}\log p\left(x_{kj}|x_{k,-j}\right)+\lambda_{j}{∥\beta_{j}∥}_{1}$$

Where *x_kj_* is the observed data for subject *k* at node *j,x_k,_*_-_*_j_* = (*x_k1,_x_k2_, …, x_k,j+1_, …, x_kn_*) and ${\text{Σ}}_{k=1}^{n}\log p\left(x_{kj}|x_{k,-j}\right)$ is the log-likelihood of all subjects. The parameter *β_j_* = *θ_j_* when *X_j_* is Gaussian; and ${\left(w_{j}^{{\left(1\right)}^{T}},w_{j}^{{\left(2\right)}^{T}},\cdot \cdot \cdot ,w_{j}^{{\left(L_{j}\right)}^{T}}\right)}^{T}$ when *X_j_* is categorical. In (1), we add an *L_1_*-penalty on the *β_j_* to enable the neighborhood selection. If node *j* is continuous, we connect node *i* with node *j* if the *i*th element of ${\hat{\beta }}_{j}$ is nonzero. If node *j* is categorical, we connect node *i* with node *j* if any *i*th element of ${\hat{w}}_{j}^{\left(k\right)}\left(k=1,\;\ldots ,L_{j}\right)\;$ is nonzero.

In the next section, we will discuss how to remove false connections identified at this stage that do not belong to the skeleton of the DAG. In (1), the tuning parameter *λ_j_* controls the level of penalization and how sparse the resulting graph will be. Its optimal value is chosen by minimizing the extended Bayesian information criteria (EBIC) (Foygel and Drton, 2010).

$$\begin{array}{l} EBIC_{\gamma }\left(\beta_{j}\right)=-2{\text{Σ}}_{k=1}^{n}\log p\left(x_{kj}|x_{k,-j}\right)+∥\beta_{j}{∥}_{0}\log n+2\gamma ∥\beta_{j}{∥}_{0}\log\left(p+q-1\right) \end{array}$$

where *n* is sample size, ∥*β*_*j*_∥_0_  is number of nonzero elements of *β_j_* and *γ* is a user-predefined constant.

### Identification of the Skeleton

The nodewise penalized GLM results in a Mixed Graphical Model (MGM), which is graphical model on continuous and discrete variables. Next, we remove edges in a MGM that do not exist in the corresponding DAG's skeleton. In a MGM, two vertices are connected if the two variables are dependent conditional on all other variables. However, in a v-structure *X* → *W* ← *Z* of a DAG, co-parents *X* and *Z* are independent conditional on their parents. Therefore, X and Z are not connected in the DAG's skeleton. But since *X* and *Z* are dependent given any vertex set that contains *W* or its descendant, *X* and *Z* are connected in a MGM. Therefore, we need to remove false connections between co-parents of v-structures in a MGM to obtain the DAG’s skeleton.

The removal of false connections between co-parents of v-structures relies on testing the conditional independence of two variables given a set of other variables. In a Gaussian graphical model, testing conditional independence is equivalent to testing a zero partial correlation coefficient (Baba et al., 2004). Therefore, such a test can be easily performed using a Fisher’s *z*-transformation (Ha et al., 2016) on the partial correlation. However, for mixed data, testing conditional independence will be more complicated as it is no longer equivalent to testing zero partial correlation coefficient. To this end, we propose a permutation method to test the conditional independence of mixed data. Let *X_j_* and *X_l_* be two variables, and *X_K_* be the set of variables that *X_j_* and *X_l_* are conditioning on. We first regress *X_j_* and *X_l_* on *X_K_* respectively using a GLM. When *X_j_* is Gaussian, we calculate the residual $r_{ij}\;=\;x_{ij}\;–{\hat{x}}_{ij},\;\left(i=1,\;\ldots ,\;n\right)\;$ from the ordinary linear regression, where *x_ij_* is the *i*th observation of *X_j_* and ${\hat{x}}_{ij}\;$ the prediction of *x_ij_* from the ordinary linear regression. When *X_j_* is discrete, we calculate the Pearson residual from a multinomial logit model

$$r_{ij}={\text{Σ}}_{k=1}^{L_{j}-1}\frac{x_{ij_{k}}-{\hat{\mu }}_{ij_{k}}}{\sqrt{{\hat{\mu }}_{ij_{k}}\left(1-{\hat{\mu }}_{ij_{k}}\right)}}$$

where *x_ijk_* the *i*th observation of the *k*th dummy variable created for *X_j_* and ${\hat{\mu }}_{ij_{k}}$ is its predicted value from the logit model. In a special case of binary outcome, the above form reduces to the Pearson residual from a logistic model. Then, we calculate the partial correlation

$${\hat{\rho }}_{jl}=\frac{{\text{Σ}}_{i=1}^{n}\left(r_{ij}-{\bar{r}}_{j}\right)\left(r_{il}-{\bar{r}}_{l}\right)}{\sqrt{{\text{Σ}}_{i=1}^{n}{\left(r_{ij}-{\bar{r}}_{j}\right)}^{2}{\text{Σ}}_{i=1}^{n}{\left(r_{il}-{\bar{r}}_{l}\right)}^{2}}}$$

where ${\bar{r}}_{j}=\frac{1}{n}{\text{Σ}}_{i=1}^{n}r_{ij}$ and$\;{\bar{r}}_{l}=\frac{1}{n}{\text{Σ}}_{i=1}^{n}r_{il}$. Next, we permute the residuals$\;{\left(r_{il}\right)}_{i=1}^{n}$ to have ${\left(r_{\pi \left(i\right)l}\right)}_{i=1}^{n}$ where *π*(*i*) ∈ {1,…,*n* } is the permuted label of *i*. The permutation is repeated for *B* times. For the *b^th^* permutation, we calculate the partial correlation

$${\hat{\rho }}_{jl}^{\left(b\right)}=\frac{{\text{Σ}}_{i=1}^{n}\left(r_{ij}-{\bar{r}}_{j}\right)\left(r_{il}^{\left(b\right)}-{\bar{r}}_{l}^{\left(b\right)}\right)}{\sqrt{{\text{Σ}}_{i=1}^{n}{\left(r_{ij}-{\bar{r}}_{j}\right)}^{2}{\text{Σ}}_{i=1}^{n}{\left(r_{il}^{\left(b\right)}-{\bar{r}}_{l}^{\left(b\right)}\right)}^{2}}}$$

The p-value testing the conditional independence of *X_j_* and *X_l_* then given by $p=\frac{1}{B}{\text{Σ}}_{i=1}^{B}I({\hat{\rho }}_{jl}>{\hat{\rho }}_{jl}^{\left(b\right)})$where *I*(*x*) is the indicator function. We conclude that *X_j_* and *X_l_* are conditionally independent if such a p-value is greater than 0.05. Based on the above test of conditional independence, we remove the edges belonging to the MGM but not the DAG's skeleton and obtain the *d*-separation set.

### Orientation of the Mixed DAG

In the last step, we add orientation to the skeleton of the DAG using a greedy search algorithm as proposed in (Tsamardinos et al., 2006). We aim to find the orientation such that the Bayesian Information Criterion (BIC) of the whole graph is minimized (Schwarz, 1978). For a given directed graph, the BIC score for the *j*th (*j* = 1,2,3,…,(*p+q*)) node is

$$BIC^{\left(j\right)}=-2\log L^{\left(j\right)}\left(\hat{\beta }\right)+∥\hat{\beta }{∥}_{0}\log n$$

where $L^{\left(j\right)}\left(\hat{\beta }\right)$ is the log-likelihood of the GLM regressing the *j*th node on its parents, $\hat{\beta }$ is the estimated vector of coefficients, and $∥\hat{\beta }{∥}_{0}$ is the number of nonzero elements in $\hat{\beta }$. The overall score of a directed graph is then given by $BIC^{\left(overall\right)}={\text{Σ}}_{j=1}^{p+q}BIC^{\left(j\right)}$. The greedy search starts from an empty graph, whose score is calculated as summation of scores of each node without any parent. Then, for a node *j* and any node *k* connected with *j* in the estimated skeleton, we attempt to add, delete or reverse an edge between them based on the BIC change. More specifically, if there is no directed edge between nodes *j* and *k* at the current iteration, we add a directed edge *j*→*k* if the BIC score becomes smaller after adding this directed edge. If there is a directed edge between nodes *j* and *k*, we delete or reverse it if the BIC score becomes smaller after deleting or reversing this edge. This algorithm stops when the above edge operations fail to decrease the overall BIC score and the resulting directed graph is the estimated DAG. For the pseudo code (**Supplementary Table S1**) and a small-scale illustration (**Supplementary Figure S1**) of our entire algorithm, see the **Supplementary Material**.

## Results

### Simulation Studies

To assess our method's performance, we simulate eight scenarios with different combinations of sample size, number of nodes and edges, and percentage of categorical nodes. We vary the sample size by 100 and 1,000; the number of nodes by 100 and 500; the percentage of categorical nodes by 10% and 20%; and the number of edges by 100 and 500. For each scenario, each categorical node contains 4 levels. More details of the simulation settings are summarized in **Table S2** in the **Supplementary Material**.

For each scenario, we first use the R package spacejam to generate a DAG. We randomly select 10% or 20% of the nodes as categorical and remaining nodes as continuous. For node *i* with no parents, if *X_i_* is continuous, *X_i_* is generated from *N*(0,1); if *X_i_* is categorical, *X_i_* is sampled from {1,2,3,4} with equal probabilities. For node *i;* with parents, if *X_i_* is continuous, *X_i_* is generated from *N*(∑_*j*∈*parent*(*i*)_*X*_*j*_, 1), where *parent* (*i*) is the parent(s) of node *i*; if *X_i_* is a categorical variable, *X_i_* is generated from *Multinomial* (1,*p*) where *p*=(*p*_1_, *p*_2_, *p*_3_, *p*_4_) and $p_{l}=\frac{\exp\left(l\sum_{j\in parent\left(i\right)}X_{j}\right)}{{\text{Σ}}_{l=1}^{4}\exp\left(l\sum_{j\in parent\left(i\right)}X_{j}\right)},l=1,2,3,4.\;$

In simulation studies, we compared our method with the CPC-stable method (implemented the R package pcalg) and the MMHC method (implemented by the R package bnlearn). Both methods cannot distinguish categorical and continuous variables but treat all of them as continuous. For each method, we evaluated edge recovery performance in both the estimated skeleton and the estimated DAG. The edge recovery performance is assessed through sensitivity, specificity, and false discovery rate (FDR). When evaluating the estimated skeleton, we define true edges as edges appearing in the true DAG's skeleton, estimated edges as edges appearing in the estimated skeleton, true null edges as unconnected edges in the true DAG's skeleton, and estimated null edges as unconnected edges in the estimated skeleton. We further defined sensitivity, specificity, and FDR of the estimated skeleton as follows:

$$Sensitivity=\frac{\#\;of\;\left[\left(estimated\;edges\cap true\;edges\right)\right]}{\#\;of\;true\;edges},$$

$$Specificity=\frac{\#\;of\;\left[estimated\;null\;edges\cap true\;null\;edges\right]}{\#\;of\;true\;null\;edges},$$

$$FDR\;=\frac{\#\;of\;\left[estimated\;edges-true\;edges\right]}{\#\;of\;estimated\;edges}$$

When evaluating the estimated DAG, we defined true edges as directed edges in the true DAG, estimated edges as directed edges in the estimated DAG, undetermined edges as edges with undetermined direction in the estimated DAG, true null edges as unconnected edges in the true DAG, and estimated null edges as unconnected edges in the estimated DAG. Then, the sensitivity, specificity, and FDR of the estimated DAG is defined as follows:

$$Sensitivity=\frac{\#\;of\;\left[\left(estimated\;edges-undermined\;edges\right)\cap true\;edges\right]}{\#\;of\;true\;edges},$$

$$Specificity=\frac{\#\;of\;\left[estimated\;null\;edges\cap true\;null\;edges\right]}{\#\;of\;true\;null\;edges},$$

$$FDR\;\left(directed\right)=\frac{\#\;of\;\left[estimated\;edges-true\;edges\right]}{\#\;of\;estimated\;edges}$$

**Figure 1:**
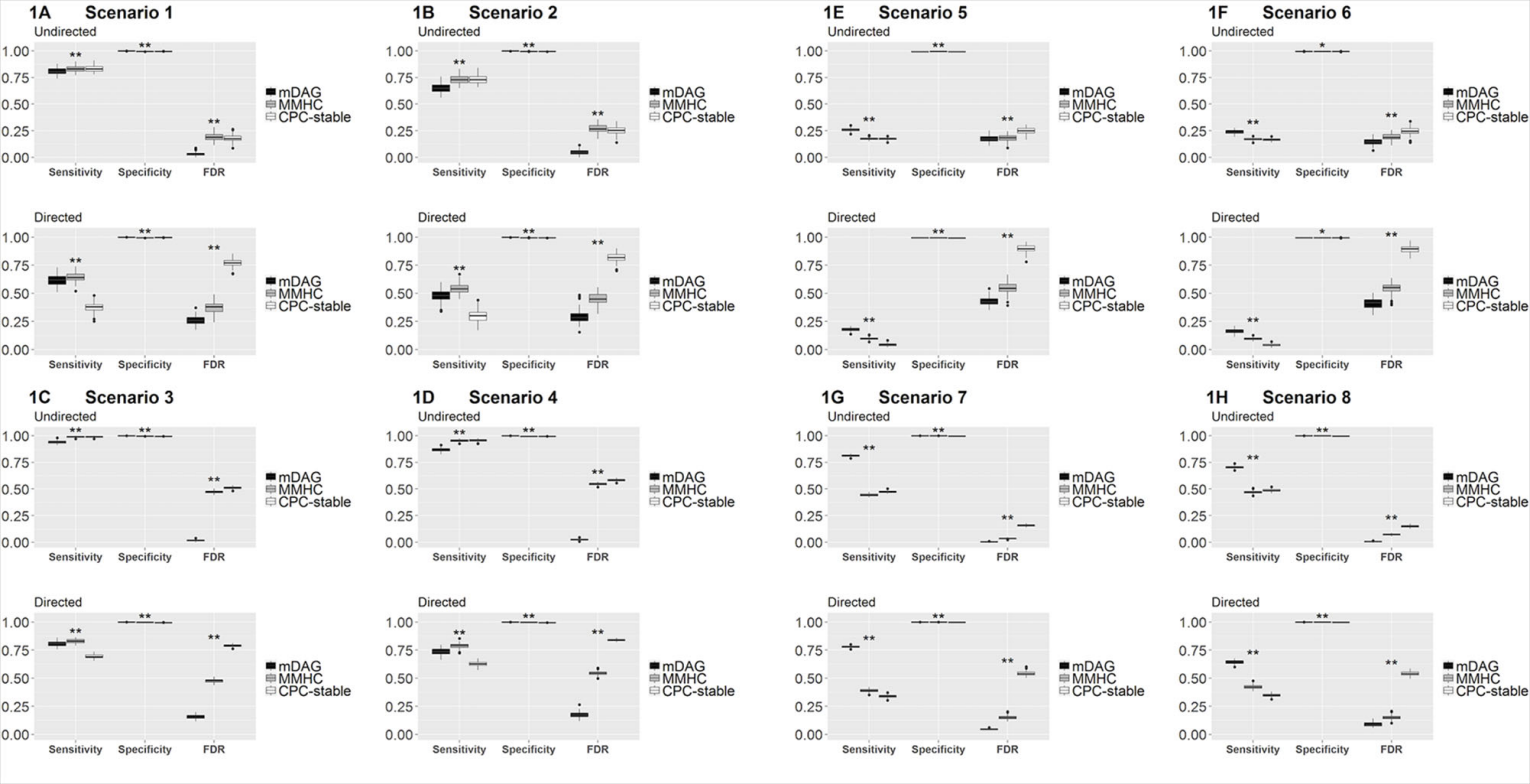
Sensitivity, specificity, and FDR of mDAG and two alternative methods, MMHC and CPC-stable, in simulation scenarios 1–8. **(A)** Scenario 1; **(B)** Scenario 2; **(C)** Scenario 3; **(D)** Scenario 4; **(E)** Scenario 5; **(F)** Scenario 6; **(G)** Scenario 7; **(H)** Scenario 8. The X-axis indicates the measurements of performance (sensitivity, specificity, and FDR); the Y-axis indicates the corresponding values. “*” indicates the sensitivity/specificity/FDR from mDAG significantly differs from the sensitivity/specificity/FDR of CPC-stable or the sensitivity/specificity/FDR of MMHC. “**” indicates the sensitivity/specificity/FDR from mDAG significantly differs from the sensitivity/specificity/FDR of CPC-stable and the sensitivity/specificity/FDR of MMHC. Such comparisons are tested by two-sample Wilcoxon.

Among the three measurements, sensitivity measures how a method recovers the connected edges in the true DAG and its skeleton. In particular, for DAG, sensitivity also measures if the direction of an edge is correctly recovered. Specificity measures how a method identifies the null edges in the true DAG and its skeleton. FDR measures the rate of falsely identified edges. In **Figure 1**, we present the boxplots of sensitivity, specificity, and FDR for all simulated scenarios.

Sensitivity, specificity, and FDR should be considered simultaneously to assess the overall edge recovery performance. In **Figures 1A–D**, the true DAG is sparse, i.e., not too many edges are connected. Our method has much better specificity and FDR for recovering the DAG and its skeleton, even though its sensitivity is smaller than the two competing methods. In **Figures 1E–H**, the true DAG is dense, i.e., many edges are connected. Our method performs the best in terms of all three measurements in both recovering the DAG and its skeleton. In all cases, our method's FDR is much lower, indicating that it estimates many fewer false positive edges. These results clearly demonstrate the merit of our methods by distinguishing categorial variables from continuous variables in the mixed data, especially when the DAG is dense. For mixed data, directly applying existing methods and ignoring data type difference clearly has inferior performance.

### Real Data Application

#### Human *Chlamydia* Infection Dataset

*Chlamydia trachomatis* can ascend from the cervix to the uterus and fallopian tubes (upper genital tract) to cause long term sequelae, including chronic pelvic pain and infertility. Inflammatory cytokines and chemokines were measured in cervical secretions from 160 asymptomatic *C. trachomatis* infected women (age 15–30 years), participating in a previously described T cell Response Against Chlamydia (TRAC) cohort (Russell et al., 2015). The Institutional Review Boards for Human Subject Research at the University of Pittsburgh and the University of North Carolina approved the study and all participants provided written informed consent prior to inclusion. Ninety-six proteins were quantified using Milliplex Magnetic Bead Assay Kits (Millipore Sigma, St. Louis, MO), as previously described (Poston et al., 2019). 160 women who were infected at enrollment were assigned to two groups: women who had both cervical and endometrial infection were defined as Endo+ (cases), while those with cervical only infection were defined as Endo- (controls). To determine the regulatory networks involved in chlamydial ascension to the endometrium, we focused on 14 cytokines that were consistently detected in cervical secretions and were tentatively positively or negatively associated with endometrial infection by univariable logistic regression after adjustment for previously determined confounders, including cervical chlamydial load and gonorrhea coinfection (*P*<0.20) (Poston et al., 2019). We jointly modeled continuous nodes, including expression of 14 cervical cytokines and one covariate (cervical chlamydial load), with categorical nodes, including the binary disease outcome (endometrial infection: Endo+ vs. Endo-) and a binary covariate (gonorrhea coinfection) by the mDAG.

**Figure 2:**
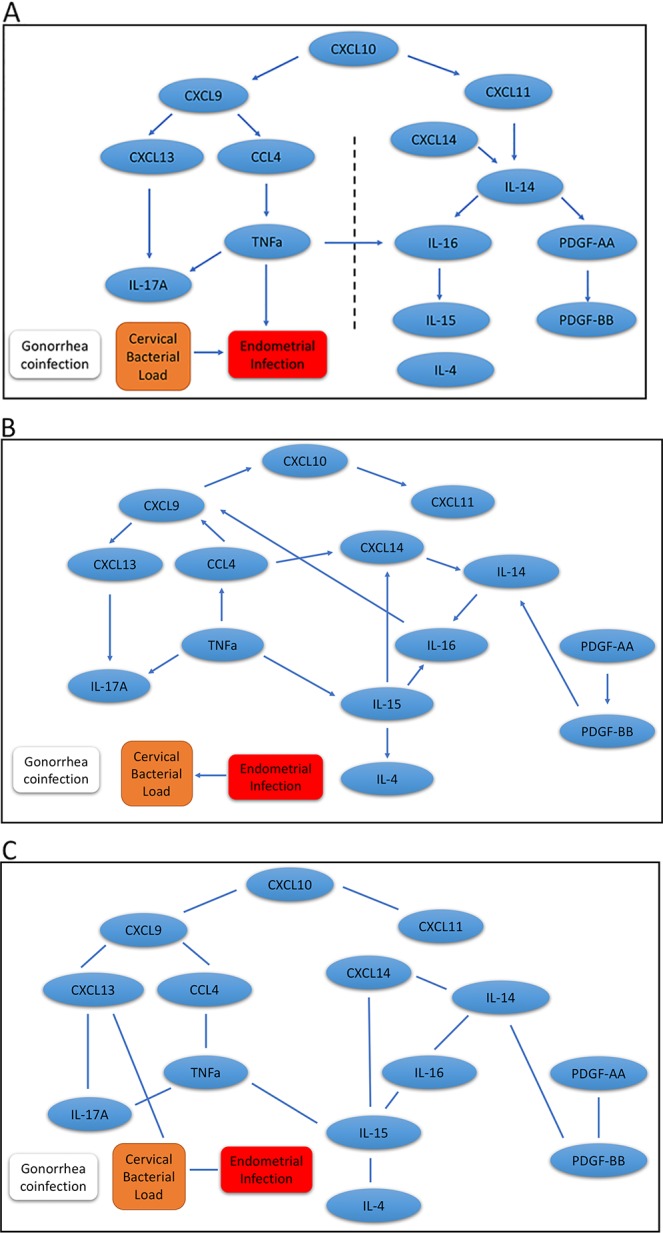
Graphic results for causal network analysis of human *Chlamydia* infection dataset, a mixed type dataset consisting of continuous variables, including expression of 14 cervical cytokines and one covariate (cervical chlamydial load), and categorical variables, including the binary disease outcome (endometrial infection: Endo+ vs. Endo-) and a binary covariate (gonorrhea coinfection) by mDAG and two alternative methods, respectively. The arrows indicate direction of causality. **(A)** mDAG; **(B)** MMHC; **(C)** CPC-stable. The dashed line in **(A)** separates cytokines connected to ascension on the left, from cytokines disconnected from ascension on the right.

Results for our mDAG analysis are shown in **Figure 2A**, and the arrows indicate direction. We found two distinct pathways that emanate from CXCL10. The CXCL9 network is connected with ascending infection, while the CXCL11 network is distant and disconnected, which indicates a more favorable host response. The CXCL9 network includes CXCL13, IL-17A, CCL4, and TNFα as downstream regulated proteins. These cytokines are predominately associated with the induction of antibody and Th17 cells that are not protective against chlamydial genital tract infection (Andrew et al., 2013; Frazer et al., 2013; Darville et al., 2019). CXCL13, a CXCR5 ligand, is produced by multiple cell types and is a potent recruiter and activator of T follicular helper (Tfh) cells and B cells (Legler et al., 1998; Breitfeld et al., 2000). CXCL13 is a marker of germinal center activity (Havenar-Daughton et al., 2016) and may also reflect increased ectopic lymphocyte cluster development (Denton et al., 2019). Thus, increased CXCL13 levels may promote or sustain plasma cell aggregates previously observed in tissues from women with chlamydial endometritis and salpingitis (Kiviat et al., 1990). Increased CXCL13 levels that stimulate plasma cell development are consistent with detection of high serum and cervical levels of anti-chlamydial IgG and IgA in women who remain susceptible to repeated chlamydial infection (Darville et al., 2019). This is consistent with the network connectivity of CXCL13 and IL-17A, since proinflammatory CXCR5+ Th17 cells are also effective B-cell helpers capable of inducing strong antibody responses (Morita et al., 2011). Furthermore, the production of TNFα by CCL4-recruited CD8 T cells may play a role in recruitment or differentiation of Th17 cells and enhance genital tract pathology (Murthy et al., 2011; Andrew et al., 2013). Besides chlamydial load, a factor we previously identified as associated with enhanced risk for upper genital tract infection, the analysis indicated TNFα production was connected with chlamydial ascension. Previous studies have linked TNFα to infertility in *C. trachomatis*-infected women (Reddy et al., 2004; Srivastava et al., 2008).

The other major network that diverges from ascension is driven by CXCL11 and includes IL-14, CXCL14, IL-16, IL-15, PDGF-AA, and PDGF-BB. CXCL11 can induce and recruit CXCR3+ T cells shown to be protective during chlamydial infection (Perry et al., 1997), and could therefore prevent ascension. CXCL11 has strong binding affinity to its receptor, CXCR3, which is consistent with the ability of CXCL11 to increase intracellular calcium at lower doses than CXCL9 (Cole et al., 1998), and may explain the deviation of these two chemokines into separate networks. Next, the convergence of CXCL14 and CXCL11 with IL-14 could represent the ability of CXCL14 to enhance CD4 T cell activation (Chen et al., 2010). This activation would lead to the release of IL-14 and subsequently stimulate local B cell activation and proliferation (Ambrus et al., 1993). Although T cell interactions with activated antigen-presenting B cells could enhance antibody production capable of initiating Fc-mediated platelet activation and PDGF release, this cell-to-cell signaling will also trigger T cell receptor-mediated IL-16 secretion (Wu et al., 1999) and further enhance CD4 T cell recruitment (Lynch et al., 2003). IL-16 can directly stimulate mononuclear phagocyte IL-15 production (Mathy et al., 2000), which is critical for T cell survival and effector function (Borger et al., 1999; Purton et al., 2007) that would protect from chlamydial ascension. These findings are consistent with our previous analysis demonstrating that cytokines downstream of CXCL9 were associated with increased odds of endometrial infection, while cytokines downstream of CXCL11 were associated with decreased odds (Poston et al., 2019).

In addition, we applied the MMHC and CPC-stable algorithms to infer the regulatory pathways. Although the MMHC (**Figure 2B**) was able to predict the causal direction among cytokines, the directionality was completely disconnected from the disease trait, and the direction between cervical bacterial load and upper genital tract infection was reversed. Regulatory networks predicted by the CPC-stable algorithm (**Figure 2C**) completely failed to infer the direction in our cytokine dataset, which might be due to its conservative feature.

These results suggest that our proposed mDAG can infer upstream causal cytokines and downstream effector cytokines more closely linked to disease and correctly separate pathogenic and protective regulatory networks.

#### Metabolic Syndrome in Men Dataset

The Metabolic Syndrome in Men (METSIM) study is a population-based study with 10,197 males randomly selected from the population register of the town of Kuopio in Finland (Stancakova et al., 2009). The Ethics Committee of the University of Eastern Finland and Kuopio University Hospital approved the METSIM study, and this study was conducted in accordance with the Declaration of Helsinki. All study participants gave written informed consent. A subset of 770 participants have gene expression measurements from subcutaneous adipose tissue (Civelek et al., 2017), we analyzed genotype, gene expression, and plasma adiponectin levels using our mDAG and alternative methods. For directional inference, we focused on two GWAS loci for adiponectin (Zhong et al., 2019) and expression of genes within ± 1Mb at each locus t. Genetic variants at the first locus near the *ADIPOQ* gene may exert their effects on adiponectin levels through expression of the *ADIPOQ* gene, which is expressed in adipose tissue and encodes the adiponectin protein studied. In contrast, genetic variants identified at the second locus, where the index SNP (the SNP with the most significant *p*-value from GWAS) is an intronic SNP in *ARL15*, which might influence adiponectin levels through expression of the *FST* gene instead of *ARL15* (Civelek et al., 2017; Martin et al., 2017; Zhong et al., 2019).

**Figure 3:**
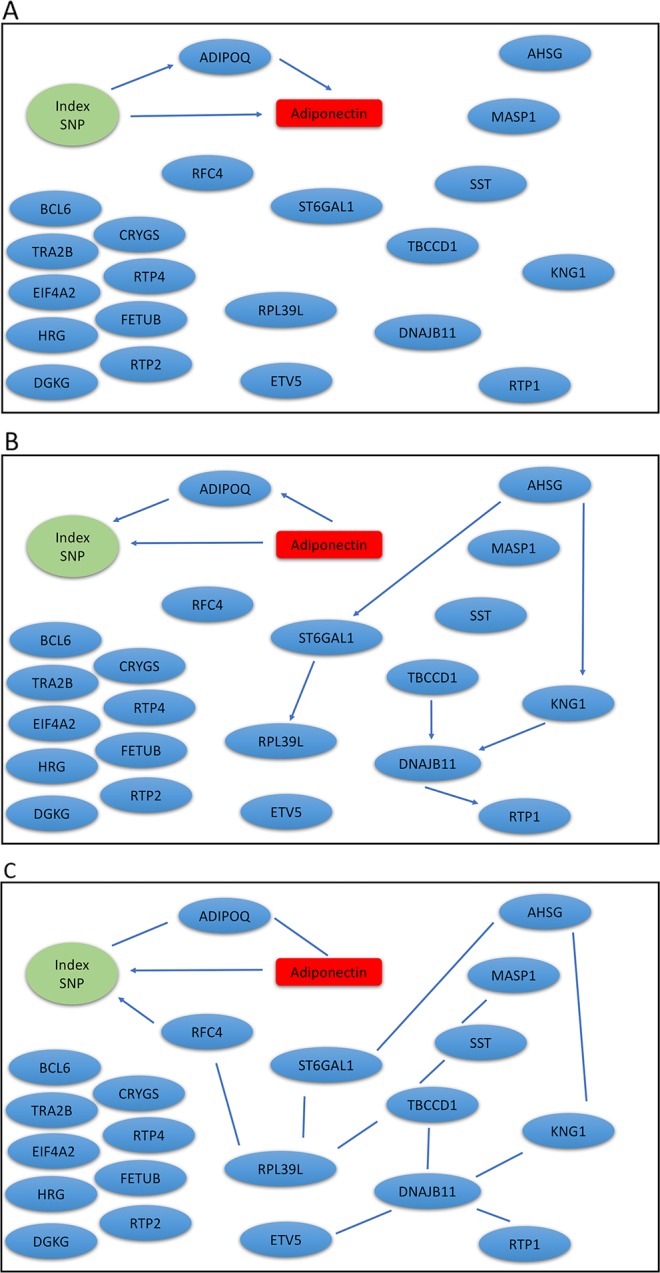
Graphic results for causal network analysis of the Metabolic Syndrome in Men dataset, a mixed type dataset consisting of a categorical variable, genotypes of one index SNP at the *ADIPOQ* GWAS locus, and several continuous variables, including expression levels of 21 genes and plasma adiponectin levels (disease trait). The arrows indicate direction of causality. **(A)** mDAG; **(B)** MMHC; **(C)** CPC-stable.

**Figure 4:**
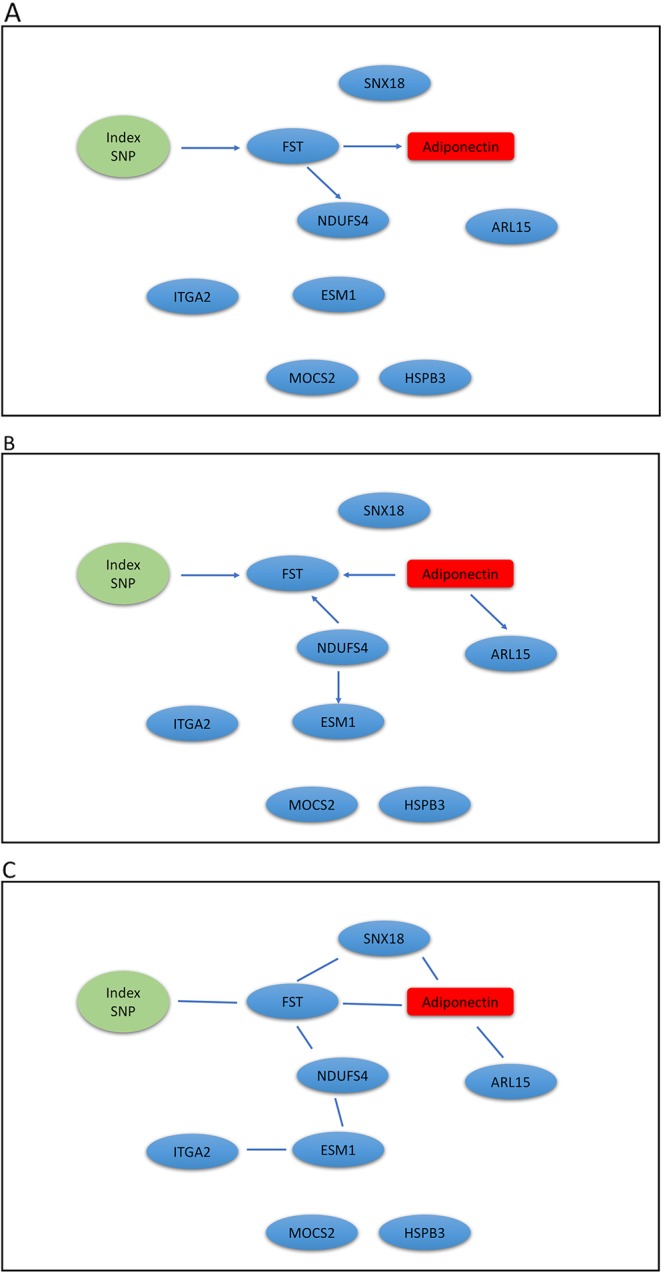
Graphic results for causal network analysis of Metabolic Syndrome in Men dataset, a mixed type dataset consisting of a categorical variable, one index SNP at *ARL15* GWAS locus, and continuous variables, including expression of 8 genes and adiponectin levels (disease trait). The arrows indicate direction of causality. **(A)** mDAG; **(B)** MMHC; **(C)** CPC-stable.

We extracted genotypes of the index SNP for each locus and expression levels of genes within ± 1Mb of each index SNP. Because a gene may have *multiple probesets*, we first applied a Sobel test to each probeset to detect mediation effect of the index SNP on adiponectin levels through the probeset. We then selected the probeset with the minimum mediation *p* value. We applied our mDAG and alternative methods to estimate DAGs (**Figures 3A–C**) for the *ADIPOQ* locus and 4A-4C for the *FST-ARL15* locus]. mDAG has the feature of forcing SNPs to point to other nodes. Results of mDAG suggest that the *ADIPOQ* gene is a mediator at the first locus (**Figure 3A**), and that *FST* gene (not *ARL15*) is a mediator at the second locus (**Figure 4A**). These findings are consistent with the results in (Zhong et al., 2019). In contrast, alternative methods failed to identify the expected directional relationships (**Figures 4B**, **C**).

## Discussion

Jointly modeling the probability distribution of the continuous measurements of gene expression or protein abundance and the categorical nodes, such as disease traits and SNPs, identifies the regulatory paths of a disease. More importantly, it distinguishes the disease-causing pathways from the disease-reaction pathways, and identifies genes mediating the effects of GWAS loci on diseases. This leads to a better understanding of disease mechanisms, and helps generate more precise targets for new therapeutic and diagnostic interventions. The existing DAG methods cannot be applied to such a joint model, as they mostly assume all nodes are of the same type.

To this end, we proposed a mixed DAG (mDAG) algorithm to infer the regulatory paths of mixed data. Our mDAG algorithm is a hybrid method and consists of three main steps including identification of the Markov blanket, determination of the skeleton, and inference of edge orientation. There are some alternative algorithms which can be applied in each step. For example, a more general framework (Zhuang et al., 2016) can be used to estimate undirected graph and PC algorithm based approach can be applied for edge orientation. Our algorithm uses a new permutation-based method to test the conditional independence of nodes of mixed types. We compared our method with two alternative well-known methods that ignore the type difference of nodes. The simulation results show that mDAG outperforms the alternative methods in terms of the FDR, sensitivity, and specificity of the edge recovery of the underlying true DAG. Results from the human chlamydial infection dataset demonstrates that mDAG successfully reconstructs the pathogenic and protective regulatory networks for chlamydial ascension. The regulatory pathways inferred by our method identify upstream causal factors and generate hypotheses for causal direction of regulatory pathways, and therefore provide candidates for experimental validation. For the Metabolic Syndrome in Men dataset, mDAG also identifies the expected paths of important GWAS loci for adiponectin suggested by previous publications (Civelek et al., 2017; Martin et al., 2017), even in the presence of multiple presumably irrelevant genes in the 1D neighborhood of the loci under study in the model, indicating that mDAG can bridge the functional gap of synonymous GWAS signals and provide the mechanistic hypotheses underlying GWAS variants.

The mDAG could not only be used to infer the causality paths in mixed types of proteomic or transcriptomic data with categorical phenotypes and/or SNP data, but it could also be applied to other mixed data, such as metabolomics and DNA structural variants, including copy number variation, since it does not require prior biological knowledge. Beyond genetics, it can be applied to social, behavioral, and psychology studies.

## Data Availability Statement

The datasets generated for this study can be found in the Gene Expression Omnibus with the accession number GSE70353.

## Ethics Statement

For the TRAC study, the Institutional Review Boards for Human Subject Research at the University of Pittsburgh and the University of North Carolina approved the study and all participants provided written informed consent prior to inclusion. For the METSIM study, the Ethics Committee of the University of Eastern Finland and Kuopio University Hospital approved the METSIM study, and this study was conducted in accordance with the Declaration of Helsinki. All study participants gave written informed consent.

## Author Contributions

Conceptualization and supervision: QL and XZ. Data curation: XZ, TD, TP, CS, KM, and YL. Resources: XZ, TD, CS, KM, and YL. Formal analysis, visualization and writing—Original draft preparation: WZ and LD. Investigation, methodology, software and validation: WZ, LD, QL, and XZ. Writing—Review and editing: QL, XZ, TD, TP, DW, KM, and YL.

## Funding

This work was supported by Development and Research Program awards by National Institutes of Health (www.nih.gov) to XZ (U19 AI144181, AI113170), National Institutes of Health (www.nih.gov) to TD (R01 AI119164, U19 AI084024 and AI007001), KM (DK093757), YL (R01 HL129132 and R01 GM105785), DL (R01 GM047845) and American Heart Association (www.heart.org) to CS (17POST33650016). The funders had no role in study design, data collection and analysis, decision to publish, or preparation of the manuscript.

## Conflict of Interest

The authors declare that the research was conducted in the absence of any commercial or financial relationships that could be construed as a potential conflict of interest.
