# Supplementary material for: Inferring Regulatory Networks From Mixed Observational Data Using Directed Acyclic Graphs
Source: Front Genet. 2020 Feb 7;11:8. doi: 10.3389/fgene.2020.00008 (PMC7038820; doi:10.3389/fgene.2020.00008)
Supplement: Supplementary file 1 [file DataSheet_1.docx]

**Supplementary Data**

**Table S1.** Pseudo code for mDAG

| **Input:** | Mixed data matrix $X$ for $d$ variables (nodes) $X=(X_{1},X_{2},\ldots,X_{d})$. Each row of $X$ is a sample, each column is a variable (node). |
| --- | --- |
| **Output:** | $d$ by $d$ matrix $G$, where $G$ represents the DAG.  $G_{ij}=1$ indicates directed edge $i\to j$; $G_{ij}=0$indicates node $i$ and $j$ are not connected. |
| **Step 1** | **1.1** For node $i$ from 1 to $d$, run the $L_{1}$-penalized GLM to identify the Markov Blanket of node $i$, with the optimal tuning parameter being chosen by EBIC.  **1.2** Define a $d$ by $d$ matrix $U$ representing an undirected graph of the $d$ variables  (nodes) $X=(X_{1},X_{2},\ldots,X_{d})$. For any pair of nodes $i$ and $j$, if node $i$ is in the Markov blanket of node $j$ or node $j$ is in the Markov blanket of node $i$, set $U_{ij}=U_{ji}=1$;  otherwise set $U_{ij}=U_{ji}=0$. |
| **Step 2** | **2.1** For any pair of nodes $i$ and $j$ with $U_{ij}=U_{ji}=1$,  if node $i$ and $j$ are marginally independent based on the permutation  test, set $U_{ij}=U_{ji}=0$.  **2.2** For any pair of nodes $i$ and $j$ with $U_{ij}=U_{ji}=1$, let  $C_{ij}$be the set of nodes that could be common children or  descendants of $i$ and $j$.  For all subsets $D_{ij}\subseteq C_{ij}$, let  $A_{ij}=\{a \vert U_{ai}=U_{ia}=1 or U_{aj}=U_{ja}=1\}$  $K=A_{ij}\backslash D_{ij}$  Test whether node$i$ and $j$ are conditional independent  given $K$using the permutation test.  If they are conditionally independent, set $U_{ij}=U_{ji}=0.$ |
| **Step 3** | **3.1** Let $G^{(old)}$ be an empty graph. Calculate its BIC score $BIC(G^{(old)})=\sum_{j=1}^{d} BIC(j)$, where $BIC(j)$ is the BIC score of node $j$based on an empty graph.  **3.2** Perform Hill Climbing greedy search algorithm to add, reverse or delete edges.  Set count=0, $\mathrm{MinScore}=BIC(G^{(old)})$  While (count<5)  For node $i$ from 1 to $d$  For node $j$ from 1 to $d$  Set $G^{(new)}= G^{(old)}$  If $G_{ij}^{(old)}=0$ and $U_{ij}=1$, set $G_{ij}^{(new)}=1$.  If $BIC(G^{\left( new \right)})>BIC(G^{(old)})$ reset $G_{ij}^{(new)}=0$  If $G_{ij}^{(old)}=1$,  Case 1: set$G_{ij}^{(new)}=0$  If $BIC(G^{\left( new \right)})>BIC(G^{\left( old \right)})$, reset $G_{ij}^{(new)}=1$  Case 2: set $G_{ji}^{(new)}=1, G_{ij}^{(new)}=0$  If $BIC(G^{\left( new \right)})>BIC(G^{(old)})$  reset $G_{ij}^{(new)}=1,G_{ji}^{(new)}=0$  If $BIC(G^{(new)})=MinScore$, then count=count+1  else set $MinScore=BIC(G^{(new)})$ |

**Table S2.** Summary of simulation scenarios.

| Scenario | Sample size | Number of nodes | Percent of categorical nodes (%) | Number of edges |
| --- | --- | --- | --- | --- |
| 1 | 100 | 100 | 10 | 100 |
| 2 | 100 | 100 | 20 | 100 |
| 3 | 1000 | 500 | 10 | 500 |
| 4 | 1000 | 500 | 20 | 500 |
| 5 | 100 | 100 | 10 | 500 |
| 6 | 100 | 100 | 20 | 500 |
| 7 | 1000 | 500 | 10 | 2500 |
| 8 | 1000 | 500 | 20 | 2500 |

Figure S1. Small-scale illustration of the mDAG algorithm. (a) True DAG; (b) Estimated MGM; (c) Estimated skeleton; (d) Estimated DAG.
